# Supplementary figures and images for: PoweREST: Statistical power estimation for spatial transcriptomics experiments to detect differentially expressed genes between two conditions
Source: PLoS Comput Biol. 2025 Jul 29;21(7):e1013293. doi: 10.1371/journal.pcbi.1013293 (PMC12316394; doi:10.1371/journal.pcbi.1013293)

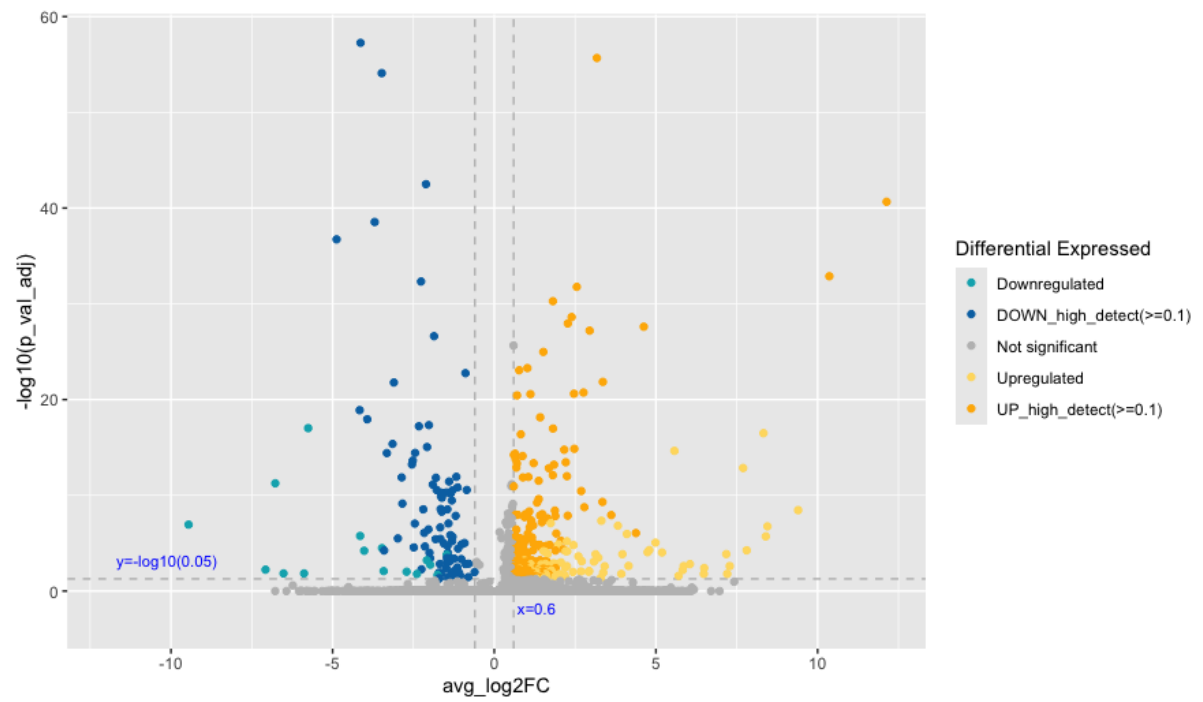

S1 Fig. Volcano plot of validation results upon IPMN dataset.

Supplement: S1 Fig — (PDF) [file pcbi.1013293.s001.pdf]

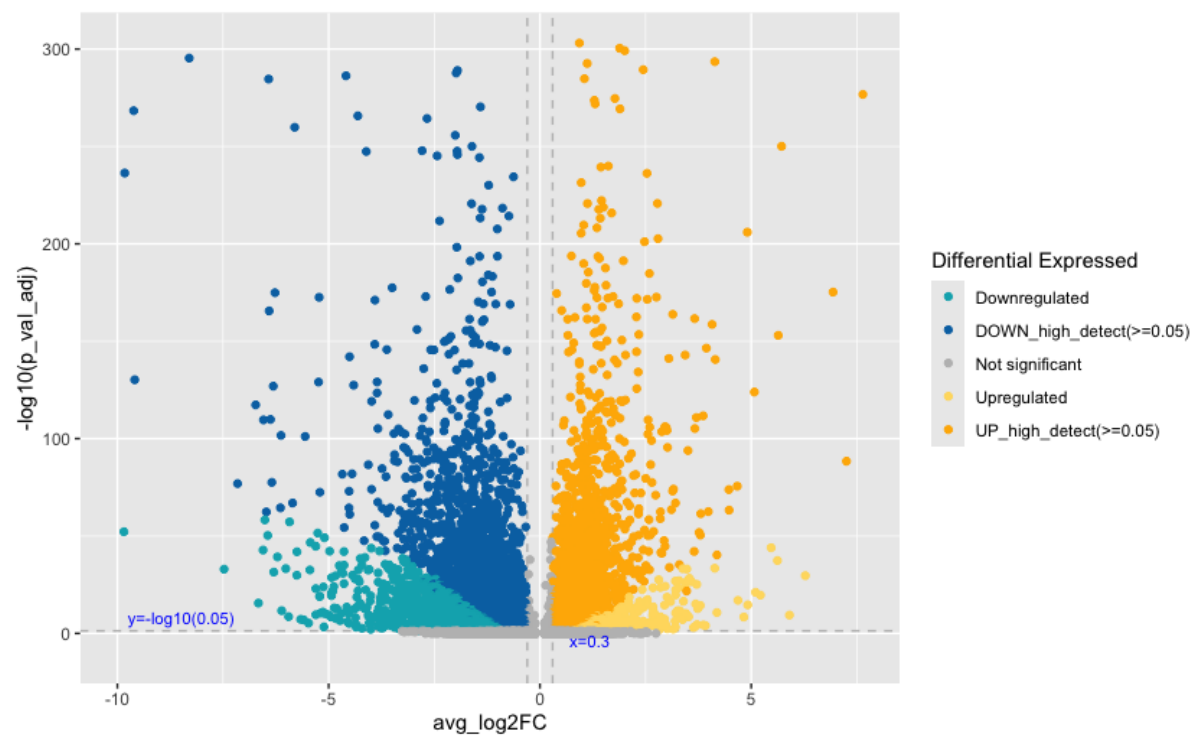

S4 Fig. Volcano plot of validation results upon CRC dataset.

Supplement: S4 Fig — (PDF) [file pcbi.1013293.s004.pdf]

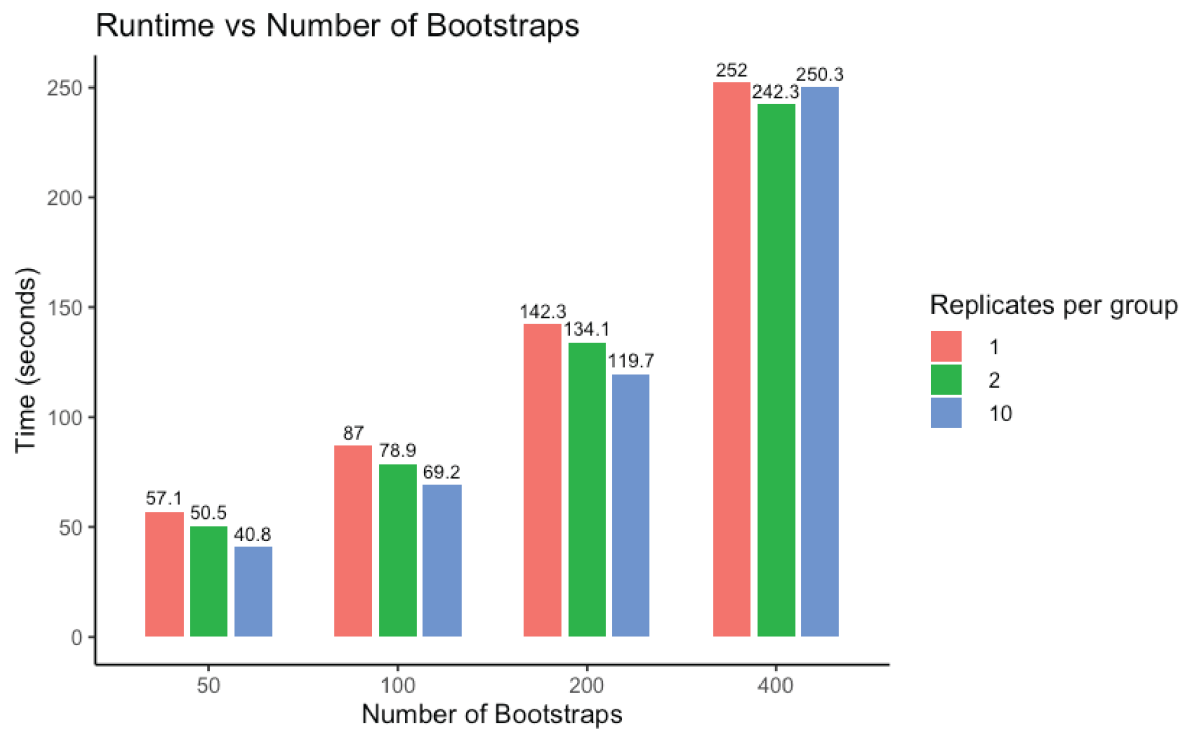

**S10 Fig. Runtime of PoweREST.** Computational time for PoweREST steps 1–3.

Supplement: S10 Fig — Computational time for PoweREST steps 1–3. (PDF) [file pcbi.1013293.s010.pdf]
