# Supplementary material for: PoweREST: Statistical power estimation for spatial transcriptomics experiments to detect differentially expressed genes between two conditions
Source: PLoS Comput Biol. 2025 Jul 29;21(7):e1013293. doi: 10.1371/journal.pcbi.1013293 (PMC12316394; doi:10.1371/journal.pcbi.1013293)
Supplement: S2 Fig — (A) Juxtalesional areas. (B) Epilesional areas. (PDF) [file pcbi.1013293.s002.pdf]

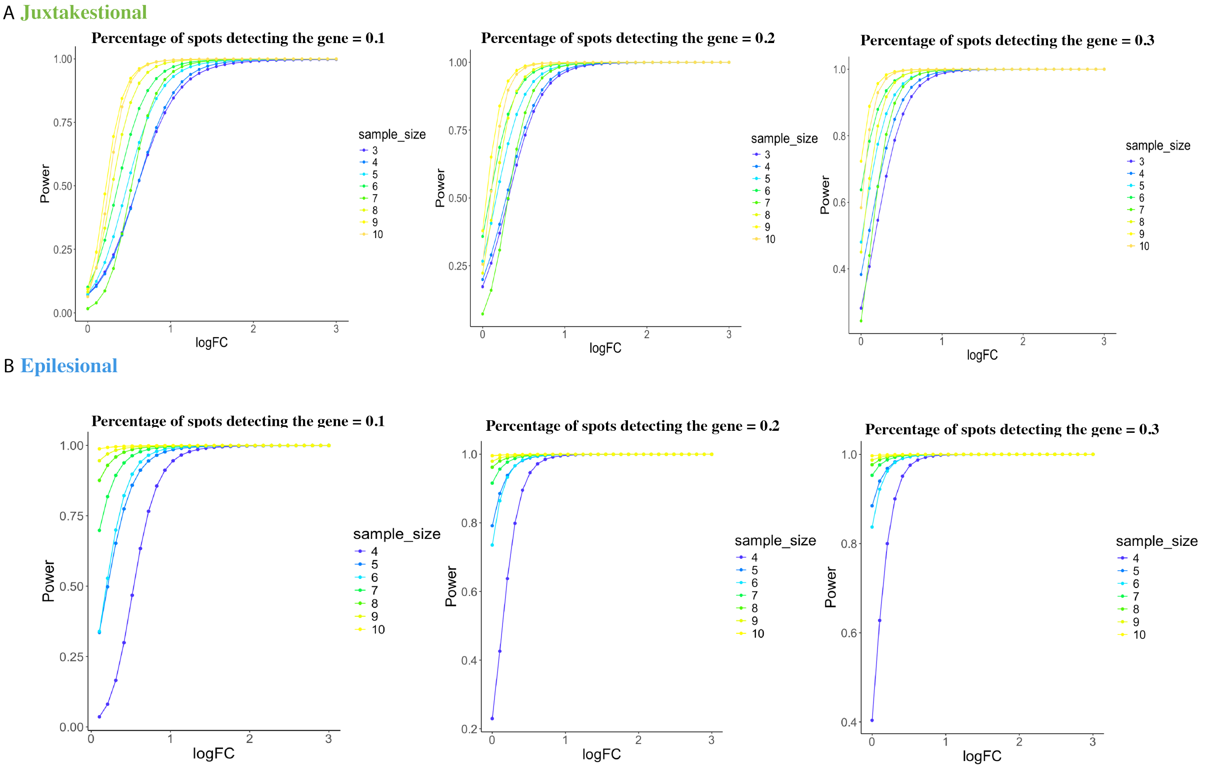

S2 Fig. The relationships between the estimated power and log fold change when the percentage of spots detecting the gene equals $0.1, 0.2, 0.3$**.** (A) Juxtalesional areas. (B) Epilesional areas.
