# Supplementary material for: PoweREST: Statistical power estimation for spatial transcriptomics experiments to detect differentially expressed genes between two conditions
Source: PLoS Comput Biol. 2025 Jul 29;21(7):e1013293. doi: 10.1371/journal.pcbi.1013293 (PMC12316394; doi:10.1371/journal.pcbi.1013293)
Supplement: S3 Fig — The relative difference between the estimated power surfaces from perilesional areas and juxtalesional areas (A), and between the estimated power surfaces from perilesional areas and epilesional areas (B), when the number of replicates per group is 6,8,10. The relative difference is calculated by comparing the difference between the estimated power values of two areas to the reference values. Specifically, it is computed using the Eq 2.Relative Difference=|Estimated Power (Juxta/Epi)−Estimated Power (Peri)|Estimated Power (Peri) (2) (PDF) [file pcbi.1013293.s003.pdf]

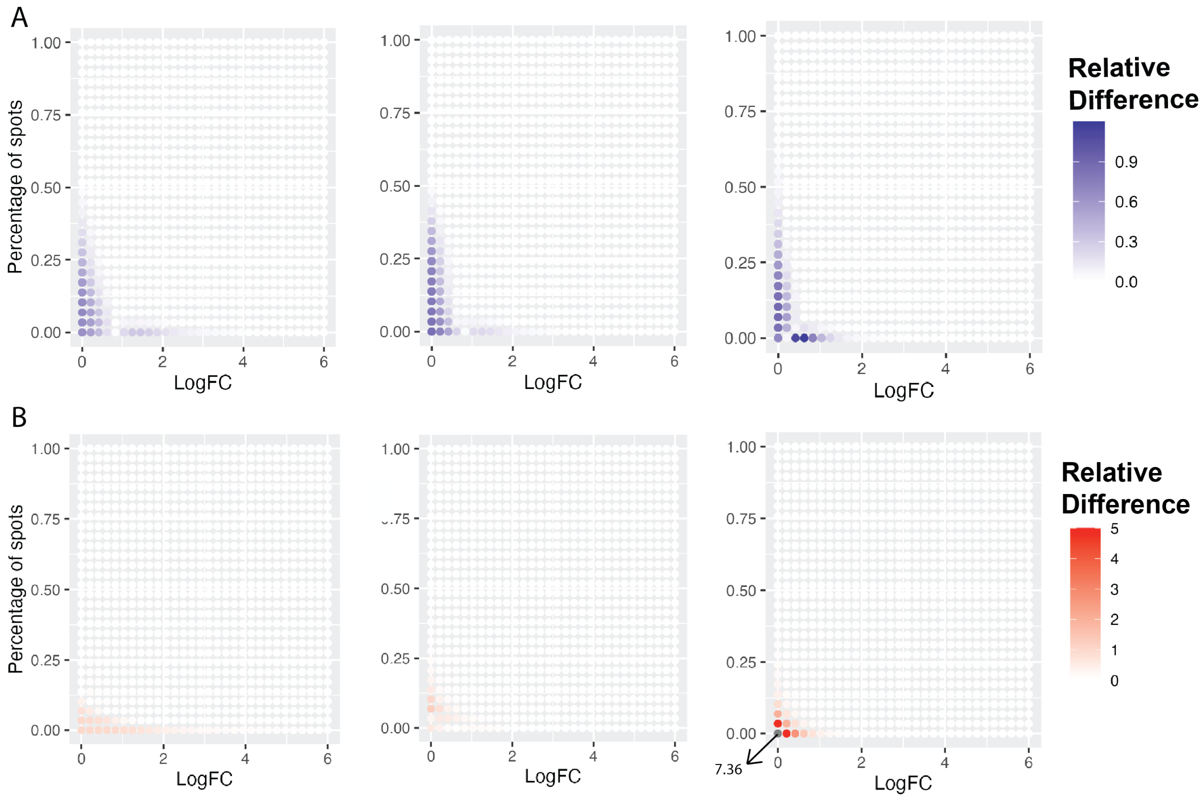

S3 Fig. The relative difference between the estimated power surfaces. The relative difference between the estimated power surfaces from perilesional areas and juxtalesional areas (A), and between the estimated power surfaces from perilesional areas and epilesional areas (B), when the number of replicates per group is 6, 8, 10. The relative difference is calculated by comparing the difference between the estimated power values of two areas to the reference values. Specifically, it is computed using the formula

$$\text{Relative Difference} = \frac{|\text{Estimated Power (Juxta/Epi)} - \text{Estimated Power (Peri)}|}{\text{Estimated Power (Peri)}} \tag{1}$$
