# Supplementary material for: PoweREST: Statistical power estimation for spatial transcriptomics experiments to detect differentially expressed genes between two conditions
Source: PLoS Comput Biol. 2025 Jul 29;21(7):e1013293. doi: 10.1371/journal.pcbi.1013293 (PMC12316394; doi:10.1371/journal.pcbi.1013293)
Supplement: S5 Fig — (PDF) [file pcbi.1013293.s005.pdf]

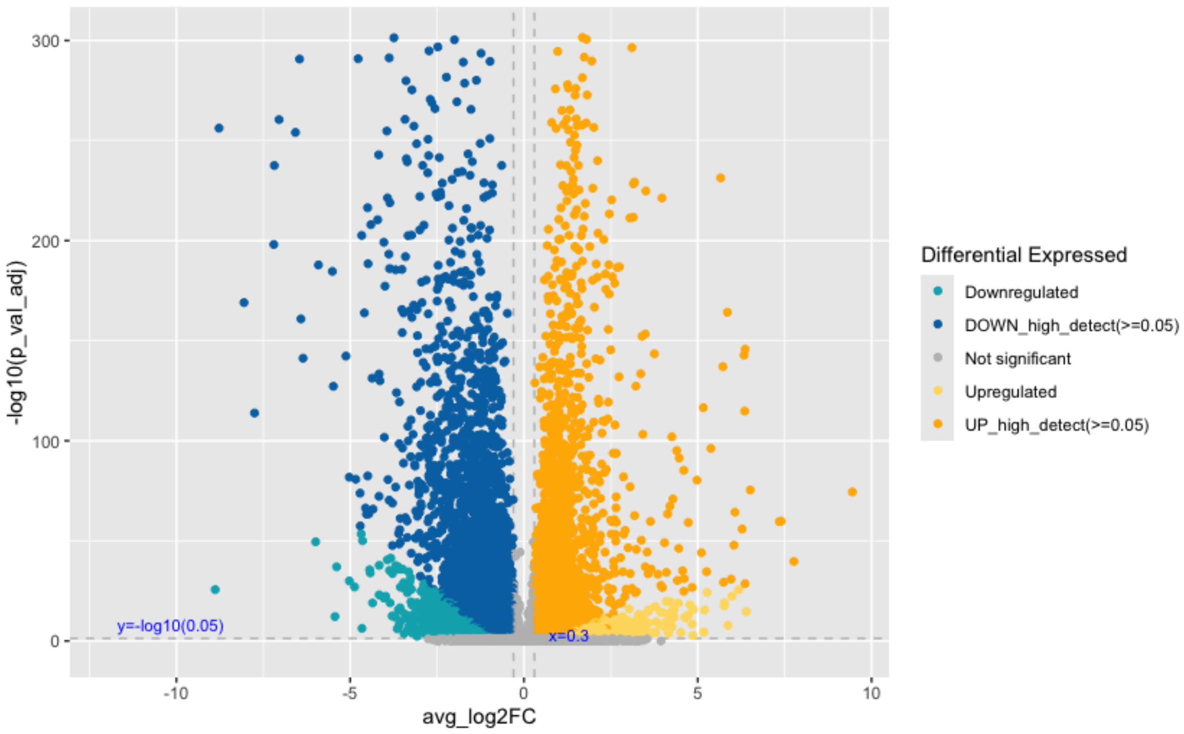


**S5 Fig. Volcano plot of validation results upon the independent CRC slices that were held out during model fitting.**
