# Supplementary material for: PoweREST: Statistical power estimation for spatial transcriptomics experiments to detect differentially expressed genes between two conditions
Source: PLoS Comput Biol. 2025 Jul 29;21(7):e1013293. doi: 10.1371/journal.pcbi.1013293 (PMC12316394; doi:10.1371/journal.pcbi.1013293)
Supplement: S6 Fig — (A) Impact of Max Depth on Root Mean Square Error (RMSE) Across Different Learning Rates and Tree Counts. (B) Impact of Learning Rate on RMSE Across Different Max Depths and Tree Counts. (PDF) [file pcbi.1013293.s006.pdf]

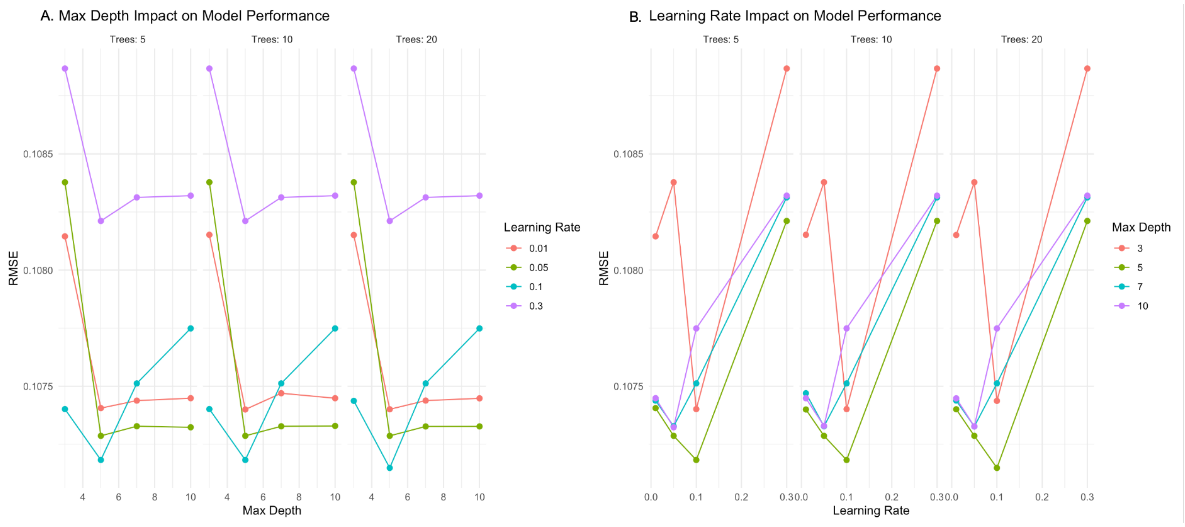


**S6 Fig. Tunning hyperparameters of XGBoost upon the validation set.** (A) Impact of Max Depth on Root Mean Square Error (RMSE) Across Different Learning Rates and Tree Counts. (B) Impact of Learning Rate on RMSE Across Different Max Depths and Tree Counts.
