# Supplementary material for: PoweREST: Statistical power estimation for spatial transcriptomics experiments to detect differentially expressed genes between two conditions
Source: PLoS Comput Biol. 2025 Jul 29;21(7):e1013293. doi: 10.1371/journal.pcbi.1013293 (PMC12316394; doi:10.1371/journal.pcbi.1013293)
Supplement: S7 Fig — (A) The fitted power surfaces for DE analysis within the carcinoma border, under the slice replicates per group being 2,4,6. A top-down 2D view is provided above each corresponding 3D surface plot. (B) The relationship between the power and logFC under slice replicates from 1 to 10, for the percentage of spots detecting the gene being 0.05,0.1,0.15. Compare with Fig 5 in the main manuscript where XGBoost was used to fit power values where logFC between 0.1 and 1 and percentage of expressed spots between 0.05 and 0.15, the estimations here are less precise due to the characteristics of XGBoost’s algorithm. (PDF) [file pcbi.1013293.s007.pdf]

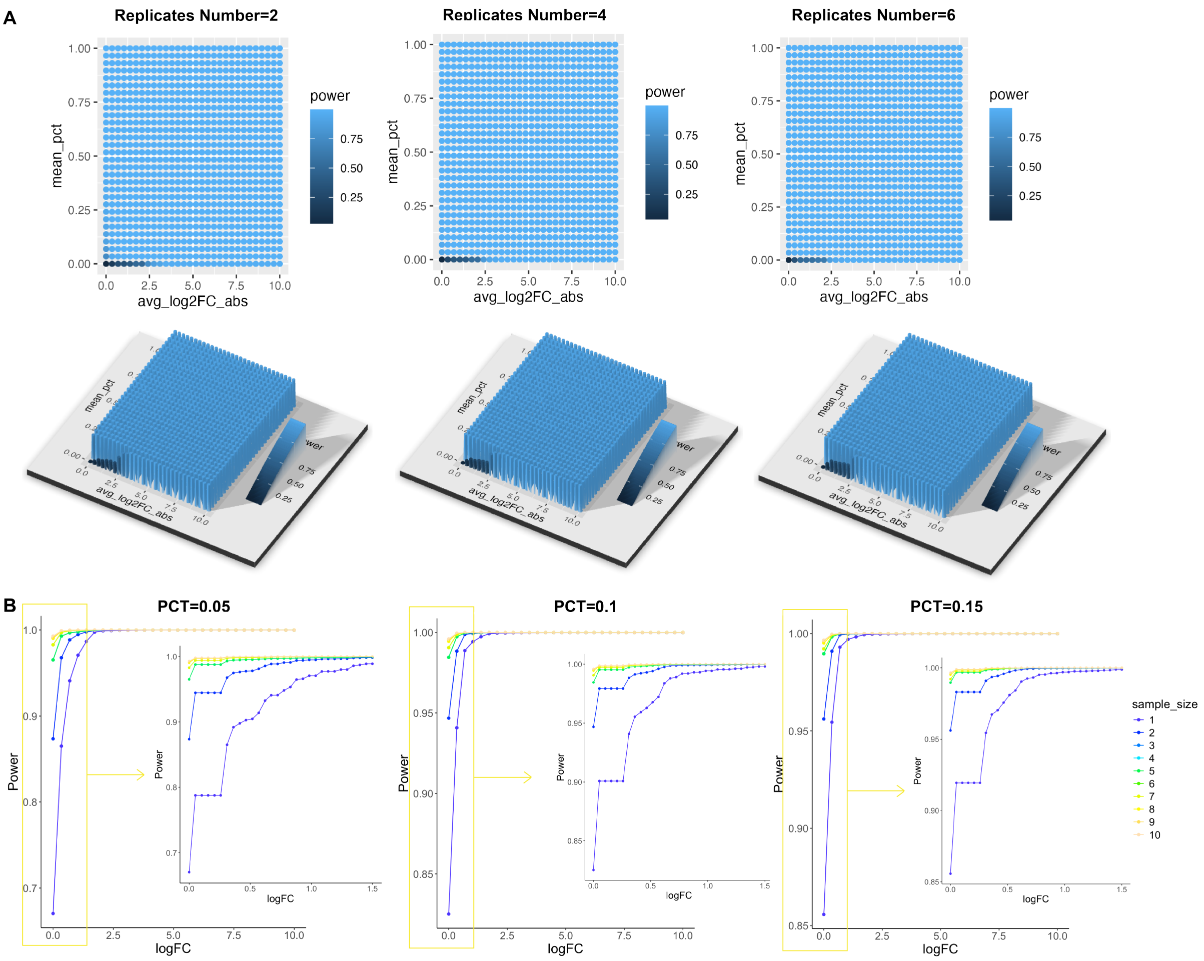

S7 Fig. Estimation upon the entire power surface using XGBoost. (A) The fitted power surfaces for DE analysis within the carcinoma border, under the slice replicates per group being $2, 4, 6$. A top-down 2D view is provided above each corresponding 3D surface plot. (B) The relationship between the power and logFC under slice replicates from 1 to 10, for the percentage of spots detecting the gene being $0.05, 0.1, 0.15$. Compare with Figure 5 in the main manuscript where XGBoost was used to fit power values where logFC between 0.1 and 1 and percentage of expressed spots between 0.05 and 0.15, the estimations here are less precise due to the characteristics of XGBoost's algorithm.
