# Supplementary material for: PoweREST: Statistical power estimation for spatial transcriptomics experiments to detect differentially expressed genes between two conditions
Source: PLoS Comput Biol. 2025 Jul 29;21(7):e1013293. doi: 10.1371/journal.pcbi.1013293 (PMC12316394; doi:10.1371/journal.pcbi.1013293)
Supplement: S8 Fig — (A) The absolute difference values between the fitting results obtained from LightGBM and XGBoost. (B) The feature importance of the fitted models. (C) The fitted XGBoost model. (D) The fitted LightGBM model. (PDF) [file pcbi.1013293.s008.pdf]

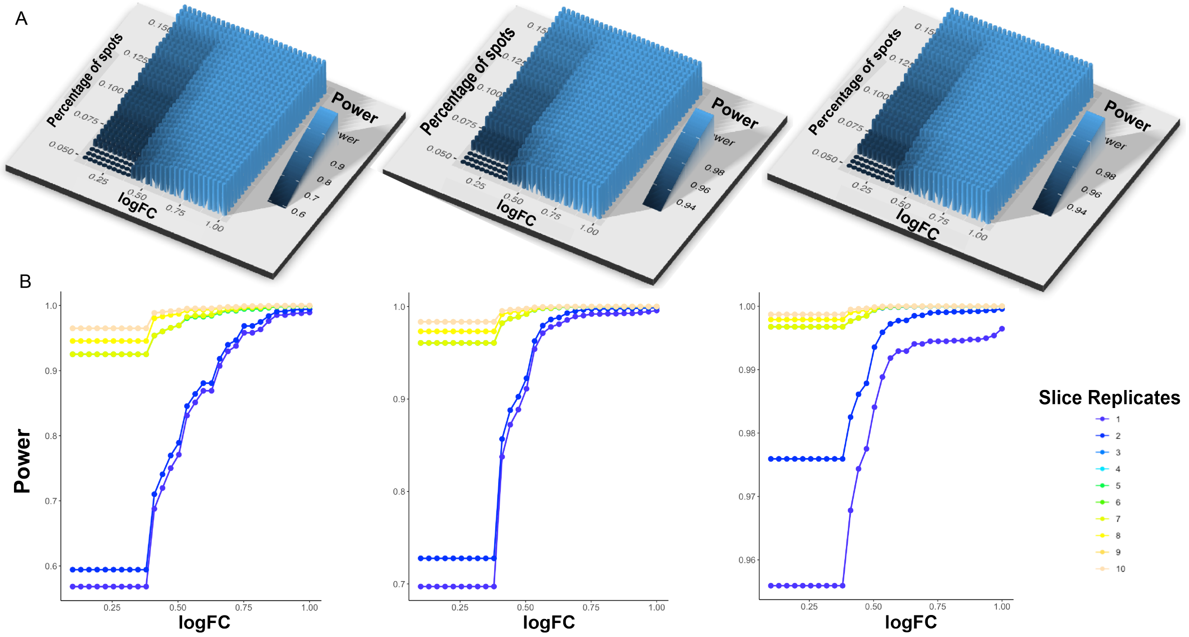

S8 Fig. LightGBM results of fitted power values where the logFC is between 0.1 and 1 and percentage of expressed spots is between 0.05 and 0.15. (A) The fitted power surfaces for DE analysis within the carcinoma border with the number of slice replicates per group being 2, 4, and 6. (B) The relationship between the power and logFC with slice replicates ranging from 1 to 10 for the percentage of spots with the detected gene being 0.05, 0.10, and 0.15.
