# Supplementary material for: PoweREST: Statistical power estimation for spatial transcriptomics experiments to detect differentially expressed genes between two conditions
Source: PLoS Comput Biol. 2025 Jul 29;21(7):e1013293. doi: 10.1371/journal.pcbi.1013293 (PMC12316394; doi:10.1371/journal.pcbi.1013293)
Supplement: S9 Fig — (A) The absolute difference values between the fitting results obtained from LightGBM and XGBoost. (B) The feature importance of the fitted models. (C) The fitted XGBoost model. (D) The fitted LightGBM model. (PDF) [file pcbi.1013293.s009.pdf]

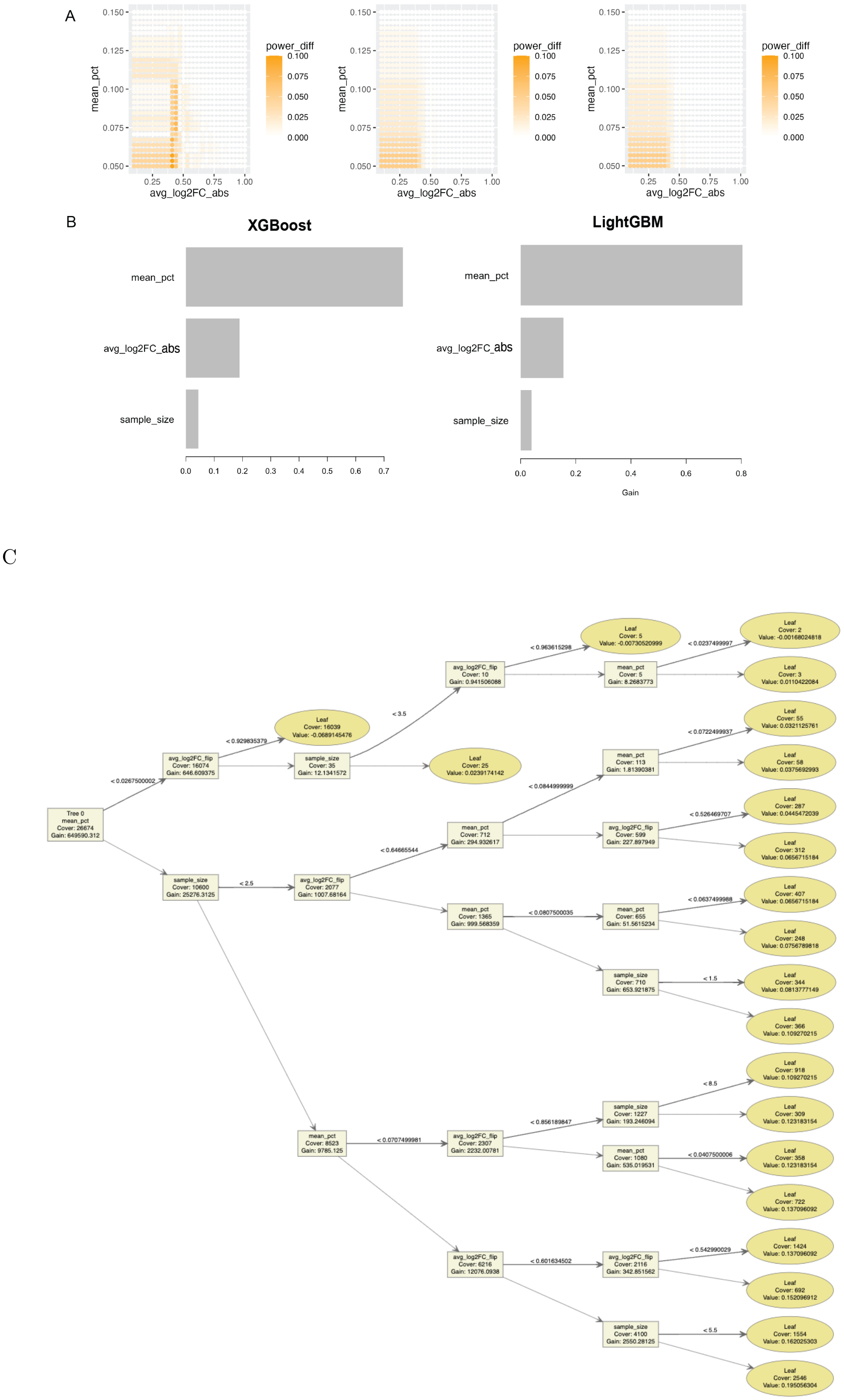
A
power_diff
mean_pct
avg_log2FC_abs
B
XGBoost
LightGBM
mean_pct
avg_log2FC_abs
sample_size
Gain
C
Tree 0
mean_pct
Cover: 26674
Gain: 649590.312
avg_log2FC_flip
Cover: 16074
Gain: 646.609375
sample_size
Cover: 10600
Gain: 25276.3125
Leaf
Cover: 16039
Value: -0.0689145476
sample_size
Cover: 35
Gain: 12.1341572
avg_log2FC_flip
Cover: 2077
Gain: 1007.68164
mean_pct
Cover: 8523
Gain: 9785.125

D

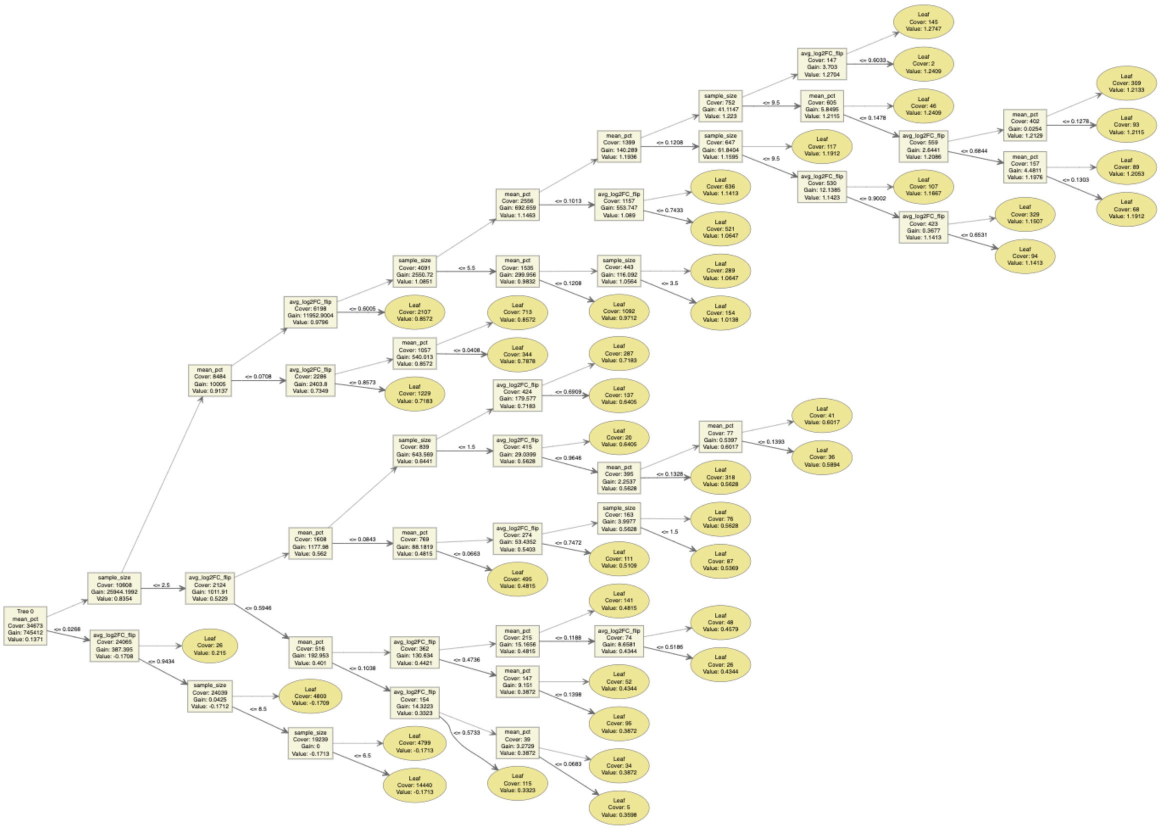

S9 Fig. Comparison between fitted results of LightGBM and XGBoost. (A) The absolute difference values between the fitting results obtained from LightGBM and XGBoost. (B) The feature importance of the fitted models. (C) The fitted XGBoost model. (D) The fitted LightGBM model.
