# Supplementary material for: PoweREST: Statistical power estimation for spatial transcriptomics experiments to detect differentially expressed genes between two conditions
Source: PLoS Comput Biol. 2025 Jul 29;21(7):e1013293. doi: 10.1371/journal.pcbi.1013293 (PMC12316394; doi:10.1371/journal.pcbi.1013293)
Supplement: S1 Table — (PDF) [file pcbi.1013293.s011.pdf]

**Gene**	**log2FC**	**Gene detection rate**	**p_val_adj**
RNF213	0.59	0.19	0.0025
ITGB2	0.63	0.19	0.0030
SEC23A	0.68	0.11	0.0400
PSMC1	0.57	0.19	0.0500

S1 Table. Four differentially expressed genes with a detection rate around 0.1 and a log-fold change around 0.6.
