# Supplementary material for: PoweREST: Statistical power estimation for spatial transcriptomics experiments to detect differentially expressed genes between two conditions
Source: PLoS Comput Biol. 2025 Jul 29;21(7):e1013293. doi: 10.1371/journal.pcbi.1013293 (PMC12316394; doi:10.1371/journal.pcbi.1013293)
Supplement: S2 Table — (PDF) [file pcbi.1013293.s012.pdf]

sample_key	patient_name	tumor_type	tumor_loc
7794_AS_3	PAT33430	MSI-H	Transverse
8578_AS_2	PAT59460	MSI-H	Ascending
7003_AS_4	PAT73458	MSI-H	Ascending
8899_AS_1	SG00001	MSI-H	Ascending
8899_AS_3	SG00002	MSI-H	Cecum
8578_AS_1	PAT01586	MSI-H	Hepatic Flexure
7794_AS_2	PAT01587	MSI-H	Ascending
6723_KL_1	PAT71397	MSS	Cecum
7003_AS_5	PAT71662	MSS	Sigmoid
7003_AS_7	PAT73899	MSS	Sigmoid
8270_AS_7	PAT30884	MSS	Sigmoid
8270_AS_9	PAT59600	MSS	Transverse
8578_AS_3	PAT59667	MSS	Cecum

S2 Table. Sample keys of slices that were included for simulation and model development.
