# Supplementary material for: PoweREST: Statistical power estimation for spatial transcriptomics experiments to detect differentially expressed genes between two conditions
Source: PLoS Comput Biol. 2025 Jul 29;21(7):e1013293. doi: 10.1371/journal.pcbi.1013293 (PMC12316394; doi:10.1371/journal.pcbi.1013293)
Supplement: S3 Table — (PDF) [file pcbi.1013293.s013.pdf]

sample_key	patient_name	tumor_type	tumor_loc
7003_AS_3	PAT73458	MSI-H	Ascending
7794_AS_1	PAT00222	MSI-H	Cecum
8270_AS_10	PAT59460	MSI-H	Ascending
8270_AS_11	PAT54273	MSI-H	Ascending
6723_KL_2	PAT71397	MSS	Cecum
7003_AS_6	PAT71662	MSS	Sigmoid
7003_AS_8	PAT73899	MSS	Sigmoid
7003_AS_1	PAT74143	MSS	Descending

S3 Table. Sample keys of slices that were held out for validation.
