# Supplementary material for: PoweREST: Statistical power estimation for spatial transcriptomics experiments to detect differentially expressed genes between two conditions
Source: PLoS Comput Biol. 2025 Jul 29;21(7):e1013293. doi: 10.1371/journal.pcbi.1013293 (PMC12316394; doi:10.1371/journal.pcbi.1013293)
Supplement: S4 Table — (PDF) [file pcbi.1013293.s014.pdf]

	**XGBoost**	**LightGBM**
How tree grows	Expand all nodes at one level before moving deeper	Expand only the best leaf
Tree shape	More balanced	More irregular and deep
Overfitting risk	Lower	Higher
**Hyperparameters**		
Step size	`eta`	`learning_rate`
Max depth	`max_depth`	`max_depth`
Max number of leaves	–	`num_leaves`
Number of parallel trees	`num_parallel_tree`	–
**Monotonic constraint**	`monotone_constraints`	`monotone_constraints`

S4 Table. Comparison between XGBoost and LightGBM decision tree growth strategies and hyperparameters.
