# Supplementary material for: PoweREST: Statistical power estimation for spatial transcriptomics experiments to detect differentially expressed genes between two conditions
Source: PLoS Comput Biol. 2025 Jul 29;21(7):e1013293. doi: 10.1371/journal.pcbi.1013293 (PMC12316394; doi:10.1371/journal.pcbi.1013293)
Supplement: S5 Table — (PDF) [file pcbi.1013293.s015.pdf]

	PoweREST	NAFLD Fibrosis Study Sample Size Design
**Data Modality**	10X Visium	NanoString GeoMx
**Parameters**	Log-fold change, gene detection rate, number of spots / number of replicates	N patients per group, N ROIs per patient, ROI diameters
**DE Test**	Supports any differential expression (DE) method	GeoDiff, glmer.nb, and GLMMadaptive with likelihood ratio test (LRT)
**Flexibility**	Generalizable framework for 10X Visium studies	Custom-built for NAFLD study, fibrosis staging, and specific DE genes (FLNA, PON1)
**Software Accessibility**	Available as an R package and R Shiny web tool	No public tool; methodology described in manuscript and supplementary files
**Monotonicity Guarantee**	Models and enforces monotonicity in the power estimation pipeline	Monotonicity not guaranteed; trends inferred empirically via spike-in calibration curves
**Spatial Awareness**	Incorporates spatial correlation implicitly via bootstrap resampling spot-level data from Visium data	Uses spot aggregation and bootstrap resampling from Visium data
**Multiple Testing Adjustment**	Includes multiple testing correction	No explicit multiple testing correction applied

S5 Table. PoweREST vs. NAFLD Fibrosis Study Sample Size Design
