# Supplementary material for: PoweREST: Statistical power estimation for spatial transcriptomics experiments to detect differentially expressed genes between two conditions
Source: PLoS Comput Biol. 2025 Jul 29;21(7):e1013293. doi: 10.1371/journal.pcbi.1013293 (PMC12316394; doi:10.1371/journal.pcbi.1013293)
Supplement: S6 Table — (A) Primary endpoint PON1 in 165 μm hepatocyte ROIs and 2 ROIs (2 Visium spots) per patient. Number of patients is indicated per group. (B) Secondary endpoint FLNA in 165 μm in fibrotic niche and 2 ROIs (2 Visium spots) per patient. Number of patients is indicated per group. (PDF) [file pcbi.1013293.s016.pdf]

A

N patients	GeoDiff		glmer.nb		GLMMadaptive LRT	
	% Power	Median (IQR) log2FC	% Power	Median (IQR) log2FC	% Power	Median (IQR) log2FC
4	27.3	1.08 (0.82–1.37)	51.0	0.33 (0.22–0.44)	38.4	0.32 (0.21–0.44)
6	35.7	0.97 (0.68–1.24)	61.9	0.33 (0.23–0.42)	54.0	0.33 (0.23–0.42)
8	37.8	0.85 (0.59–1.13)	74.5	0.33 (0.26–0.41)	68.7	0.33 (0.25–0.41)
10	44.3	0.78 (0.53–1.06)	82.5	0.33 (0.26–0.40)	78.2	0.33 (0.26–0.40)

B

N patients	GeoDiff		glmer.nb		GLMMadaptive LRT	
	% Power	Median (IQR) log2FC	% Power	Median (IQR) log2FC	% Power	Median (IQR) log2FC
4	33.4	0.83 (0.56–1.13)	72.7	1.04 (0.80–1.22)	60.4	1.02 (0.77–1.21)
6	39.9	0.82 (0.57–1.09)	84.0	1.04 (0.84–1.22)	76.2	1.04 (0.83–1.21)
8	46.2	0.81 (0.59–1.03)	91.0	1.03 (0.85–1.19)	86.6	1.02 (0.84–1.17)
10	54.9	0.81 (0.61–1.07)	93.5	1.03 (0.88–1.16)	91.4	1.02 (0.88–1.16)

S6 Table. Final simulation results from NAFLD fibrosis sample size design. (A) Primary endpoint PON1 in 165 μm hepatocyte ROIs and 2 ROIs (2 Visium spots) per patient. Number of patients is indicated per group. (B) Secondary endpoint FLNA in 165 μm in fibrotic niche and 2 ROIs (2 Visium spots) per patient. Number of patients is indicated per group.
