# Supplementary material for: PoweREST: Statistical power estimation for spatial transcriptomics experiments to detect differentially expressed genes between two conditions
Source: PLoS Comput Biol. 2025 Jul 29;21(7):e1013293. doi: 10.1371/journal.pcbi.1013293 (PMC12316394; doi:10.1371/journal.pcbi.1013293)
Supplement: S7 Table — Estimated power values across varying log-fold changes and gene detection rates, stratified by number of slices per group (fixed at 80 spots per slice). (PDF) [file pcbi.1013293.s017.pdf]

Gene detection rate	Log-fold change				
	0.3	0.6	0.9	1.2	1.5
**4 slices per group, 80 spots per slice**					
0.1	0.36	0.76	0.90	0.94	0.96
0.2	0.64	0.92	0.97	0.99	0.99
0.3	0.83	0.98	0.99	1.00	1.00
0.4	0.93	0.99	1.00	1.00	1.00
0.5	0.97	1.00	1.00	1.00	1.00
**6 slices per group, 80 spots per slice**					
0.1	0.63	0.92	0.97	0.98	0.99
0.2	0.86	0.99	1.00	1.00	1.00
0.3	0.97	1.00	1.00	1.00	1.00
0.4	0.99	1.00	1.00	1.00	1.00
0.5	1.00	1.00	1.00	1.00	1.00
**8 slices per group, 80 spots per slice**					
0.1	0.74	0.95	0.98	0.99	0.99
0.2	0.92	0.99	1.00	1.00	1.00
0.3	0.97	1.00	1.00	1.00	1.00
0.4	0.99	1.00	1.00	1.00	1.00
0.5	1.00	1.00	1.00	1.00	1.00
**10 slices per group, 80 spots per slice**					
0.1	0.84	0.97	0.98	0.99	1.00
0.2	0.96	0.99	1.00	1.00	1.00
0.3	0.99	1.00	1.00	1.00	1.00
0.4	1.00	1.00	1.00	1.00	1.00
0.5	1.00	1.00	1.00	1.00	1.00

S7 Table. PoweREST's power estimations for IPMN's perilesional areas. Estimated power values across varying log-fold changes and gene detection rates, stratified by number of slices per group (fixed at 80 spots per slice).
