# Supplementary material for: PoweREST: Statistical power estimation for spatial transcriptomics experiments to detect differentially expressed genes between two conditions
Source: PLoS Comput Biol. 2025 Jul 29;21(7):e1013293. doi: 10.1371/journal.pcbi.1013293 (PMC12316394; doi:10.1371/journal.pcbi.1013293)
Supplement: S8 Table — (PDF) [file pcbi.1013293.s018.pdf]

	**PoweREST**	**RnaSeqSampleSize[1]**	**POWSC[2]**
**Data Modality**	10X Visium	Bulk RNA-seq	Single-cell RNA-seq
**Parameters**	Log-fold change, gene detection rate, number of spots / number of replicates	Read counts, dispersion, fold-change, sample size	Number of cells, sequencing depths; four gene-wise variables (mean, variance, coefficient of variation, zero fraction) and two cell-wise variables (library size, zero fraction)
**Spatial Awareness**	**Supports spot-level modeling and spatial structure**	**No**; assumes independence of gene expression	**No**; assumes that gene expression is independent between cells
**Monotonicity Guarantee**	Models and enforces monotonicity in the power estimation pipeline	Monotonicity not guaranteed	Monotonicity not guaranteed
**Multiple Testing Adjustment**	Includes multiple testing correction	Includes multiple testing correction	Includes multiple testing correction

S8 Table. PoweREST vs. Power estimation methods developed for bulk and single-cell RNA-seq data.

References

[1] Zhao S, Li CI, Guo Y, Sheng Q, Shyr Y. RnaSeqSampleSize: real data based sample size estimation for RNA sequencing. BMC bioinformatics. 2018 Dec;19:1-8.

[2] Su K, Wu Z, Wu H. Simulation, power evaluation and sample size recommendation for single-cell RNA-seq. Bioinformatics. 2020 Oct 1;36(19):4860-8.
