# Supplementary material for: PoweREST: Statistical power estimation for spatial transcriptomics experiments to detect differentially expressed genes between two conditions
Source: PLoS Comput Biol. 2025 Jul 29;21(7):e1013293. doi: 10.1371/journal.pcbi.1013293 (PMC12316394; doi:10.1371/journal.pcbi.1013293)
Supplement: S1 Appendix — (PDF) [file pcbi.1013293.s019.pdf]

S1 Appendix. Intuition upon 3D monotonic relationships from the mathematical formula. Derived by Noether [1], the approximate power formula for the Wilcoxon rank sum test in the DE analysis on gene expression X and Y in the first and second group can be expressed as:

$$Power \approx 1 - \Phi(z_{\frac{\alpha}{2}} + \sqrt{6n_{xy}}(\frac{1}{2} - Prob(Y > X))), \tag{2}$$

where Φ is the cumulative distribution function for the standard normal $N(0,1)$ and $z_{\frac{\alpha}{2}}$ is the alpha-level z-score for a two-tailed test. When the Wilcoxon rank sum test is performed using the synthetic specimen ST data, n_{xy} is the product of spots number n, target replicates number N, and percentage of spots detecting the gene π_g. Intuitively, $Prob(Y > X)$ can be influenced by the log fold-change β_g. Thus, from the formula, we can infer that the study power grows with an increased absolute value of β_g or increased expression rate π_g. However, the actual power formula is hard to define without further assumptions regarding the gene expression data. For this reason, we chose bootstrap resampling to estimate the power. Moreover, our nonparametric methodology can readily accommodate power calculations for various DE tests.

References

[1] Noether GE. Sample size determination for some common nonparametric tests. Journal of the American Statistical Association. 1987 Jun 1;82(398):645-7.
