# Supplementary material for: PoweREST: Statistical power estimation for spatial transcriptomics experiments to detect differentially expressed genes between two conditions
Source: PLoS Comput Biol. 2025 Jul 29;21(7):e1013293. doi: 10.1371/journal.pcbi.1013293 (PMC12316394; doi:10.1371/journal.pcbi.1013293)
Supplement: S2 Appendix — (PDF) [file pcbi.1013293.s020.pdf]

S2 Appendix. P-spline fitting under 2D monotonic constraints. Our aim is to construct 2D smooth, $m(\pi_g, |\beta_g|)$. Consider marginal smooth functions for π_g and $|\beta_g|$:

$$f_1(\pi_g) = \sum_{k_1=1}^{q_1} \alpha^1_{k_1} B_{k_1}(\pi_g), \tag{3}$$

$$f_1(|\beta_g|) = \sum_{k_2=1}^{q_2} \alpha^2_{k_2} B_{k_2}(|\beta_g|), \tag{4}$$

where B_{k_1} and B_{k_2} are B-spline, $\alpha^1_{k_1}$ and $\alpha^2_{k_2}$ are spline coefficients. Then $m(\pi_g, |\beta_g|)$ may be written as follows:

$$m(\pi_g, |\beta_g|) = \sum_{k_1=1}^{q_1} \sum_{k_2=1}^{q_2} B_{k_1}(\pi_g) B_{k_2}(|\beta_g|) \gamma_{k1k2}, \tag{5}$$

where γ_{k1k2} are unknown coefficients. Therefore, we can write equation(5) in matrix form:

$$\boldsymbol{m} = \boldsymbol{X\gamma}, \tag{6}$$

where $\mathbf{X}$ is the matrix such that its ith row is $\mathbf{X_i} = \mathbf{X_{1i}} \otimes \mathbf{X_{2i}}$, $\mathbf{X_1}$ is the matrix form of $B_{k1}(\pi_g)$, $\mathbf{X_2}$ is the matrix form of $B_{k2}(|\beta_g|)$ and $\otimes$ denotes the Kronecker product.

It is well known that a sufficient condition for monotonic constraints $\partial m(\pi_g, |\beta_g|)/\pi_g \geq 0$ is $\gamma_{k_1k_2} \geq \gamma_{(k_1-1)k_2}$. To impose these conditions, Pya and Wood [1] proposed the re-parametrization $\boldsymbol{\gamma} = \boldsymbol{\Sigma}\tilde{\boldsymbol{\beta}}$, where $\tilde{\boldsymbol{\beta}} = [\beta_{11}, ..., exp(\beta_{k_1k_2}), ..., exp(\beta_{q_1q_2})]^T$ and $\boldsymbol{\Sigma} = \boldsymbol{\Sigma_1} \otimes \boldsymbol{\Sigma_2}$ where

$$\Sigma_{1,ab} = \begin{cases} 0 \text{ when } a < b \\ 1 \text{ when } a \geq b \end{cases} \tag{7}$$

and same for $\boldsymbol{\Sigma_2}$.

A smoothing penalty is further imposed upon $\boldsymbol{\beta}$:

$$\text{Penalty} = \lambda_1 \boldsymbol{\beta}^T \boldsymbol{S_1} \boldsymbol{\beta} + \lambda_2 \boldsymbol{\beta}^T \boldsymbol{S_2} \boldsymbol{\beta}, \tag{8}$$

where $\boldsymbol{S_1} = \boldsymbol{D_1}^T \boldsymbol{D_1}$ and $\boldsymbol{D_1} = \boldsymbol{D_{m1}} \otimes \boldsymbol{I_2}$. $\boldsymbol{D_{m1}}$ is the $(q_1 - 2) \cdot q_1$ matrix that is all zero except that $D_{i,i+1} = -D_{i,i+2} = 1$ for $i = 1, ..., q_1 - 2$ and $\boldsymbol{I_2}$ is an identity matrix of size q_2. $\boldsymbol{S_2} = \boldsymbol{D_2}^T \boldsymbol{D_2}$ and $\boldsymbol{D_2} = \boldsymbol{I_1} \otimes \boldsymbol{D_{m2}}$. $\boldsymbol{D_{m2}}$ is the $(q_2 - 2) \cdot q_2$ matrix that is all zero except that $D_{i,i+1} = -D_{i,i+2} = 1$ for $i = 1, ..., q_2 - 2$ and $\boldsymbol{I_1}$ is an identity matrix of size q_1.

Pya and Wood [1] applied the Newton-Raphson method to maximize the penalized likelihood for estimation of the $\boldsymbol{\gamma}$. The estimations are robust to the choice of q_1 and q_2 when shape constraints are employed [2].

References

[1] Pya N, Wood SN. Shape constrained additive models. Statistics and computing. 2015 May;25:543-59.

[2] Meyer MC. Constrained penalized splines. Canadian Journal of Statistics. 2012 Mar;40(1):190-206.
