# Supplementary material for: PoweREST: Statistical power estimation for spatial transcriptomics experiments to detect differentially expressed genes between two conditions
Source: PLoS Comput Biol. 2025 Jul 29;21(7):e1013293. doi: 10.1371/journal.pcbi.1013293 (PMC12316394; doi:10.1371/journal.pcbi.1013293)
Supplement: Attachment — Submitted filename: Response_letter.docx [file pcbi.1013293.s021.docx]

We thank the editor and reviewers for their thorough reviews, thoughtful comments and constructive suggestions for our manuscript “**PoweREST: Statistical Power Estimation for Spatial Transcriptomics Experiments to Detect Differentially Expressed Genes Between Two Conditions**” (PCOMPBIOL-D-25-00403). We appreciate the positive comments such as “particularly important” and “highlights the importance of power analysis in ST studies”.

Based on the editor’s and reviewers’ comments, we have carefully revised the manuscript (modifications in the manuscript are highlighted in blue font). In this revision, we made the following major modifications:

1. Conducted additional validation analysis on the power calculation results of both IPMN and CRC datasets.
2. Reserved a subset of CRC tissue slices from the original dataset, which were held out during model development, and used them to further validate the power estimation results.
3. Performed a technical and numerical comparison between PoweREST with NanoString GeoMx power estimation method, as well as existing methods for bulk and single-cell RNA-seq data.
4. Performed the model fitting using LightGBM, an alternative machine learning algorithm based on the gradient boosting framework and obtained results consistent with those from XGBoost.
5. Evaluated the computational performance for PoweREST simulation pipeline under varying numbers of bootstrap iterations and replicates per group.

We addressed the individual points raised by the reviewers, as detailed below. The reviewers’ comments are in *italic blue*, and our replies are in regular font.

**Response to Editor Comments:**

*The reviewers have raised important concerns about your work, all of which must be addressed before the paper can be accepted. In particular, it is crucial to clearly position your work against previous approaches for power estimation. In addition, both reviewers have raised key concerns about the technical details of your approach and its applicability to different types of spatial transcriptomic data.*

Answer: We thank the editor for this helpful comment. In the revised manuscript, we have addressed these two key concerns by taking the following modifications:

1. We have performed both technical and numerical comparisons between PoweREST and the NanoString GeoMx power estimation method, as well as existing methods for bulk and single-cell RNA-seq data. The details of these comparisons, including methodology and results, are provided in our response to **Reviewer 1’s Comment 3** and are also reflected in the revised manuscript.

2. We stated under **Reviewer 1’s Comment 1** and **Reviewer 2’s Comment 5** that though our current implementation of PoweREST is tailored to the 10X Genomics Visium platform, the core methodological framework of PoweREST is general and extensible. In future work, we plan to generalize PoweREST to support other ST platforms.

**Response to Reviewer #1’s comments:**

1. *The manuscript focuses exclusively on 10X Genomics Visium data, which uses fixed 55-µm spots. However, other ST platforms (e.g., Slide-seq, MERFISH, Seq-Scope, or NanoString GeoMx) have different spatial resolutions, data structures, and ROI selection methods. The tool's applicability to these platforms is not discussed.*

**Answer:** We thank the reviewer for the helpful comment. We agree that spatial transcriptomics technologies vary significantly in spatial resolution, data structure, and ROI selection methods. These platform-specific characteristics indeed influence the statistical modeling assumptions required for power estimation. Because of this, for each platform, different power calculation models should be designed with cautions.

Our current implementation of PoweREST is tailored to the 10X Genomics Visium platform, due to its widespread adoption and lack of corresponding power calculation tools. However, the core methodological framework of PoweREST is general and extensible.

To address this point, we have added

Though PoweREST is designed for 10X Genomics Visium platform and was evaluated on the visium data in this study, its non-parametric framework is inherently flexible and can be extend to other ST platforms, as its simulation-based approach does not assume platform-specific parametric distributions. In future work, we plan to generalize PoweREST to support other ST platforms.

in the Discussion section (page 12, lines 369-373) briefly mentioning how our method can potentially be adapted for other platforms.

2. *The manuscript validates PowerREST using two publicly available datasets (IPMN and CRC). While this is a good start, the tool's performance on independent datasets or experimental validation (e.g., comparing predicted power with observed power in new experiments) is lacking.*

**Answer:** We appreciate the reviewer’s thoughtful comment regarding the need for further validation of PowerREST. As suggested, we have further validated our power estimation results by performing differential expression (DE) analysis on the two datasets originally used in the manuscript (IPMN and CRC) as well as on an independent subset of CRC tissue slices that was held out prior to any simulation or model development. This held-out CRC dataset was not used in training or parameter tuning and thus serves as an independent validation of the power estimation results.

For the Peri area of IPMN dataset, where PoweREST estimated a statistical power of **0.92** with **6** slices per group for detecting a DE gene characterized by a detection rate of **0.1** and an absolute log-fold change of **0.6**. To validate this, we randomly selected 6 slices from each group of the IPMN dataset and performed DE analysis. The results, shown in Figure 1 (also available as S1 Fig in Supplementary material), confirm our model's power assessment. Specifically, we identified **four differentially expressed genes** with detection rates close to 0.1 and absolute log-fold changes approximately 0.6 (Table 1, also available as S1 Table in Supplementary material). In the Results section (page 7, lines 243-248), we added

We further validated the power estimation results by randomly selecting subsets of slices from the IPMN dataset and comparing observed DE results to the predicted power. Specifically, for the Peri region, with 6 slices per group, we predicted a statistical power of 0.92 for detecting a DE gene with a detection rate of 0.1 and an absolute log-fold change of 0.6. Our validation results (S1 Fig and S1 Table) confirm this power assessment.

**Figure 1**: Volcano plot of validation results upon IPMN dataset.

**Table 1**: Four differentially expressed genes with a detection rate around 0.1 and a log-fold change around 0.6.

|  | **log2FC** | **Gene detection rate** | **p_val_adj** |
| --- | --- | --- | --- |
| RNF213 | 0.59 | 0.19 | 0.0025 |
| ITGB2 | 0.63 | 0.19 | 0.003 |
| SEC23A | 0.68 | 0.11 | 0.04 |
| PSMC1 | 0.57 | 0.19 | 0.05 |

For the carcinoma border region of the CRC dataset, PoweREST estimated that with **4** slices per group, the power to detect a gene with a detection rate of **0.05** and an absolute log-fold change of **0.3** would be **0.91**. To validate this power estimation, we randomly selected 4 slices from each group among the CRC slices used for simulation and performed DE analysis. The results, shown in Figure 2 (also available as S4 Fig in Supplementary material), support our estimation. Specifically, we identified a DE gene, named *IGHV6-1*, with an average detection rate of 0.017 (<0.05), an average log-fold change of -0.12 (0.12<0.3), and an adjusted p-value being 2.2e-05, based on the randomly selected 4 slices per group.

**Figure 2**: Volcano plot of validation results upon CRC dataset.

We further validated this power estimation using independent CRC slices that were initially held out prior to the simulation process. The sample keys for the slices included in the simulation and those held out for independent validation are listed in Tables 2-3 (also available as S2-3 Tables in Supplementary material). The results based on 4 slices from each group among these independent CRC slices are shown in Figure 3 (also available as S5 Fig in Supplementary material). Specifically, apart from *IGHV6-1*, we identified another differentially expressed gene, called *IGHD*, with an average detection rate of 0.02 (<0.05), an average log-fold change of 0.29 (<0.3), and an adjusted p-value being 0.0004.

**Table 2**: sample keys of slices included for simulation and model development. These are samples we initially used in our previous manuscript.

| sample_key | patient_name | tumor_type | tumor_loc |
| --- | --- | --- | --- |
| 7794_AS_3 | PAT33430 | MSI-H | Transverse |
| 8578_AS_2 | PAT59460 | MSI-H | Ascending |
| 7003_AS_4 | PAT73458 | MSI-H | Ascending |
| 8899_AS_1 | SG00001 | MSI-H | Ascending |
| 8899_AS_3 | SG00002 | MSI-H | Cecum |
| 8578_AS_1 | PAT01586 | MSI-H | Hepatic Flexure |
| 7794_AS_2 | PAT01587 | MSI-H | Ascending |
| 6723_KL_1 | PAT71397 | MSS | Cecum |
| 7003_AS_5 | PAT71662 | MSS | Sigmoid |
| 7003_AS_7 | PAT73899 | MSS | Sigmoid |
| 8270_AS_7 | PAT30884 | MSS | Sigmoid |
| 8270_AS_9 | PAT59600 | MSS | Transverse |
| 8578_AS_3 | PAT59667 | MSS | Cecum |

**Table 3**: sample keys of slices that were held out for validation.

| sample_key | patient_name | tumor_type | tumor_loc |
| --- | --- | --- | --- |
| 7003_AS_3 | PAT73458 | MSI-H | Ascending |
| 7794_AS_1 | PAT00222 | MSI-H | Cecum |
| 8270_AS_10 | PAT59460 | MSI-H | Ascending |
| 8270_AS_11 | PAT54273 | MSI-H | Ascending |
| 6723_KL_2 | PAT71397 | MSS | Cecum |
| 7003_AS_6 | PAT71662 | MSS | Sigmoid |
| 7003_AS_8 | PAT73899 | MSS | Sigmoid |
| 7003_AS_1 | PAT74143 | MSS | Descending |

**Figure 3**: Volcano plot of validation results upon the independent CRC slices.

We included the above validation results for CRC dataset in the Result section (page 9, lines 271-276)

The dataset contains 31 human colonic specimens with about 17,000 genes detected. From this dataset, we selected 7 tissue samples diagnosed as microsatellite instability-high (MSI-H) CRC, 6 tissue samples diagnosed as microsatellite stable (MSS) CRC for simulation and model development, while holding out the remaining slices for independent validation. The sample keys for the slices included in the simulation and those for validation are listed in S2-S3 Tables.

and

We further validated the power estimation results by randomly selecting subsets of slices from those used for simulation and comparing observed DE results to the predicted power. Specifically, with 4 slices per group, we predicted a statistical power of 0.91 for detecting a DE gene with a detection rate of 0.05 and an absolute log-fold change of 0.3. We also performed the validation using independent slices that were initially held out. Both results (S4-S5 Figs) confirm the power assessment of the proposed method.

in the Result section (page 10, lines 294-300).

3. *The manuscript briefly mentions the NanoString GeoMx power estimation method but does not provide a detailed comparison with PowerREST. A more thorough comparison with existing methods (such as for single-cell, bulk RNA-seq data) would highlight the unique advantages and limitations of PowerREST.*

**Answer:** We thank the reviewer for this insightful suggestion. We agree that a more comprehensive comparison with existing power estimation methods would help clarify the distinct contributions of PowerREST.

In response, we conducted a detailed comparison between PowerREST and NAFLD Fibrosis Study Sample Size Design specialized for **NanoString GeoMx**^[1]^ (Tables 4-7, also available as S5-S7 Tables in Supplementary material), as well as other relevant approaches developed for **bulk and single-cell RNA-seq data**, specifically RnaSeqSampleSize^[2]^ and POWSC^[3]^ (Table 8, also available as S8 Table in Supplementary material). Briefly, compared with the NanoString GeoMx power estimation method^[1]^, our method is more generalizable, ensures monotonicity and supports multiple testing correction. Compared with existing approaches for bulk and single-cell RNA-seq data, our method incorporates the spatial structure by bootstrapping the spot-level gene expressions within the predefined ROI, while RnaSeqSampleSize^[2]^ focuses on bulk data without the ability to address cellular or near-cellular transcriptomic measurements and POWSC^[3]^  assume independence between cells (in single-cell RNA-seq). Therefore, none of the existing methods can account for spatial autocorrelation or spot-level heterogeneity present in ST datasets.

We added

Compared with the existing method developed for NanoString GeoMx in the NAFLD fibrosis study, PoweREST offers both technical and numerical advantages in flexibility, accessibility, and power estimation performance. While the NAFLD framework is tailored to a specific application, staging liver fibrosis based on two selected genes (e.g., PON1 and FLNA), PoweREST provides a generalizable and non-parametric approach that supports any differential expression method, spatial structure, and allows user-defined input parameters such as gene detection rate and log-fold change. We summarized the detailed comparison in S5-S7 Tables.

in the Discussion Section (page 12, paragraph 3, lines 361-369)

and

Compared with existing approaches developed for bulk and single-cell RNA-seq data, our method uniquely incorporates spatial structure by bootstrapping spot-level gene expression within predefined ROIs, whereas prior methods either lack the resolution to address near-cellular measurements or assume independence between individual cells, limiting their applicability to spatial transcriptomics data (S8 Table).

in the Discussion Section (page 12, lines 375-380).

**Table 4**: PoweREST vs. NAFLD Fibrosis Study Sample Size Design^[1]^

|  | PoweREST | NAFLD Fibrosis Study Sample Size Design |
| --- | --- | --- |
| Data Modality | 10X Visium | NanoString GeoMx |
| Parameters | Log-fold change, gene detection rate, number of spots number/ number of replicates | N patients per group, N ROIs per patient, ROI diameters |
| DE Test | Supports any differential expression (DE) method | GeoDiff, glmer.nb and GLMMadaptive with likelihood ratio test (LRT) |
| Flexibility | Generalizable framework for 10X Visium studies | Custom-built for NAFLD study, fibrosis staging, and specific DE genes (FLNA, PON1) |
| Software Accessibility | Available as an R package and R Shiny web tool | No public tool; methodology described in manuscript and supplementary files |
| Monotonicity Guarantee | Models and enforces monotonicity in the power estimation pipeline | Monotonicity not guaranteed; trends inferred empirically via spike-in calibration curves |
| **Spatial Awareness** | **Incorporates spatial correlation implicitly via bootstrap resampling spot-level data from Visium data** | **Uses spot aggregation and bootstrap resampling from Visium data** |
| Multiple Testing Adjustment | Includes multiple testing correction | No explicit multiple testing correction applied |

**Table 5**: Final simulation results from NAFLD fibrosis sample size design^[1]^: primary endpoint PON1 in 165 μm hepatocyte ROIs and 2 ROIs (2 Visium spots) per patient. Number of patients is indicated per group.

|  | GeoDiff |  | glmer.nb |  | GLMMadaptive LRT |  |
| --- | --- | --- | --- | --- | --- | --- |
| N patients | % Power | Median (IQR) log2FC | % Power | Median (IQR) log2FC | % Power | Median (IQR) log2FC |
| 4 | 27.3 | 1.08 (0.82–1.37) | 51 | 0.33 (0.22–0.44) | 38.4 | 0.32 (0.21–0.44) |
| 6 | 35.7 | 0.97 (0.68–1.24) | 61.9 | 0.33 (0.23–0.42) | 54.0 | 0.33 (0.23–0.42) |
| 8 | 37.8 | 0.85 (0.59–1.13) | 74.5 | 0.33 (0.26–0.41) | 68.7 | 0.33 (0.25–0.41) |
| 10 | 44.3 | 0.78 (0.53–1.06) | 82.5 | 0.33 (0.26–0.4) | 78.2 | 0.33 (0.26–0.4) |

**Table 6**: Final simulation results from NAFLD fibrosis sample size design^[1]^: Secondary endpoint FLNA in 165 μm in fibrotic niche and 2 ROIs (2 Visium spots) per patient. Number of patients is indicated per group.

|  | GeoDiff |  | glmer.nb |  | GLMMadaptive LRT |  |
| --- | --- | --- | --- | --- | --- | --- |
| N patients | % Power | Median (IQR) log2FC | % Power | Median (IQR) log2FC | % Power | Median (IQR) log2FC |
| 4 | 33.4 | 0.83 (0.56–1.13) | 72.7 | 1.04 (0.8–1.22) | 60.4 | 1.02 (0.77–1.21) |
| 6 | 39.9 | 0.82 (0.57–1.09) | 84 | 1.04 (0.84–1.22) | 76.2 | 1.04 (0.83–1.21) |
| 8 | 46.2 | 0.81 (0.59–1.03) | 91 | 1.03 (0.85–1.19) | 86.6 | 1.02 (0.84–1.17) |
| 10 | 54.9 | 0.81 (0.61–1.07) | 93.5 | 1.03 (0.88–1.16) | 91.4 | 1.02 (0.88–1.16) |

**Table 7**: PoweREST’s slimuation results for IPMN dataset

| Log-fold change | 0.3 | 0.6 | 0.9 | 1.2 | 1.5 |
| --- | --- | --- | --- | --- | --- |
| Gene detection rate | Power | | | | |
| **10 slices per group, 80 spots per slices** | | | | | |
| 0.1 | 0.84 | 0.97 | 0.98 | 0.99 | 1 |
| 0.2 | 0.96 | 0.99 | 1 | 1 | 1 |
| 0.3 | 0.99 | 1 | 1 | 1 | 1 |
| 0.4 | 1 | 1 | 1 | 1 | 1 |
| 0.5 | 1 | 1 | 1 | 1 | 1 |
| **8 slices per group, 80 spots per slices** | | | | | |
| 0.1 | 0.74 | 0.95 | 0.98 | 0.99 | 0.99 |
| 0.2 | 0.92 | 0.99 | 1 | 1 | 1 |
| 0.3 | 0.97 | 1 | 1 | 1 | 1 |
| 0.4 | 0.99 | 1 | 1 | 1 | 1 |
| 0.5 | 1 | 1 | 1 | 1 | 1 |
| **6 slices per group, 80 spots per slices** | | | | | |
| 0.1 | 0.63 | 0.92 | 0.97 | 0.98 | 0.99 |
| 0.2 | 0.86 | 0.99 | 1 | 1 | 1 |
| 0.3 | 0.97 | 1 | 1 | 1 | 1 |
| 0.4 | 0.99 | 1 | 1 | 1 | 1 |
| 0.5 | 1 | 1 | 1 | 1 | 1 |
| **4 slices per group, 80 spots per slices** | | | | | |
| 0.1 | 0.36 | 0.76 | 0.90 | 0.94 | 0.96 |
| 0.2 | 0.64 | 0.92 | 0.97 | 0.99 | 0.99 |
| 0.3 | 0.83 | 0.98 | 0.99 | 1 | 1 |
| 0.4 | 0.93 | 0.99 | 1 | 1 | 1 |
| 0.5 | 0.97 | 1 | 1 | 1 | 1 |

**Table 8**: PoweREST vs. Power estimation methods developed for bulk and single-cell RNA-seq data

|  | PoweREST | RnaSeqSampleSize^[2]^ | POWSC^[3]^ |
| --- | --- | --- | --- |
| Data Modality | 10X Visium | Bulk RNA-seq | Single-cell RNA-seq |
| Parameters | Log-fold change, gene detection rate, number of spots number/ number of replicates | Read counts, dispersion, fold-change, sample size | Number of cells, sequencing depths, (four gene-wise variables): mean, variance, coefficient of variation and zero fraction; (two cell-wise variables): library size and zero fraction. |
| **Spatial Awareness** | **Supports spot-level modeling and spatial structure** | **No; assumes independence of gene expression** | **No; assumes that gene expression is independent between cells** |
| Monotonicity Guarantee | Models and enforces monotonicity in the power estimation pipeline | Monotonicity not guaranteed | Monotonicity not guaranteed |
| Multiple Testing Adjustment | Includes multiple testing correction | Includes multiple testing correction | Includes multiple testing correction |

[1] Ryaboshapkina, M. and Azzu, V., 2023. Sample size calculation for a NanoString GeoMx spatial transcriptomics experiment to study predictors of fibrosis progression in non-alcoholic fatty liver disease. Scientific Reports, 13(1), p.8943.

[2] Zhao, S., Li, C.I., Guo, Y., Sheng, Q. and Shyr, Y., 2018. RnaSeqSampleSize: real data based sample size estimation for RNA sequencing. BMC bioinformatics, 19, pp.1-8.

[3] Su, K., Wu, Z. and Wu, H., 2020. Simulation, power evaluation and sample size recommendation for single-cell RNA-seq. Bioinformatics, 36(19), pp.4860-4868.

4. *While the manuscript demonstrates the application of PowerREST to IPMN and CRC datasets, it does not fully explore the biological or clinical implications of the findings.*

**Answer:** We appreciate the reviewer’s comment and agree that discussing the biological and clinical relevance of our findings would enhance the impact of the manuscript. In the revised version, we have added

PoweREST is an essential tool as it can assist researchers in optimizing ST study design by avoiding both over-sampling and under-sampling during the early planning stages.

in the Introduction section (page 3, lines 73-74) emphasizing that our power calculation framework can assist researchers in optimizing study design by avoiding both over-sampling (which increases cost) and under-sampling (which compromises statistical validity). While the primary focus of this work is methodological, additional biological insights related to the IPMN and CRC datasets used in our study can be found in the original publications from which these datasets were derived.

5. *While XGBoost is proposed as a solution for maintaining monotonicity, it is a machine learning model that can overfit the data, especially when applied to small or noisy datasets. This could lead to unreliable power estimates in some cases.*

**Answer:** We thank the reviewer for highlighting this important consideration. We fully acknowledge the potential for overfitting when using machine learning models such as XGBoost, particularly in the context of small or noisy datasets. Our XGBoost model was trained on the data comprising over 30,000 data points, which is a substantial sample size for XGBoost-based model development. We partitioned the dataset into 80% for training, 10% for validation, and 10% for testing. To prevent overfitting, we employed cross-validation and early stopping during model training. Additionally, we controlled model complexity by tuning hyperparameters, including maximum tree depth (max_depth $\epsilon c(3, 5,7,10)$), number of parallel trees (number of parallel trees $\epsilon c(5,10,15)$), and learning rate (learning rate $\epsilon c(0.01, 0.05, 0.1, 0.3)$), based on the performance on the validation set. As shown in Figure 4 (also available as S6 Fig in Supplementary material), we found that the optimal maximum tree depth is 5, and the optimal learning rate is 0.1. However, as the Root Mean Squared Error (RMSE) values vary only slightly across different numbers of parallel trees, we chose to set it to 5 to simplify the model. The final RMSE on the test set is 0.12, which is close to the RMSE values got on the validation set. We added

To prevent possible overfitting, we partitioned the dataset into 80% for training, 10% for validation, and 10% for testing and employed cross-validation and early stopping during model training. Additionally, we controlled model complexity by tuning hyperparameters, including maximum tree depth, number of parallel trees and learning rate based on the performance on the validation set (S6 Fig).

to the Result section (page 11, lines 304-308).

**Figure 4**: Tunning hyperparameters of XGBoost upon the validation set. (A) Impact of Max Depth on Root Mean Square Error (RMSE) Across Different Learning Rates and Tree Counts. (B) Impact of Learning Rate on RMSE Across Different Max Depths and Tree Counts.

6. *The manuscript demonstrates PowerREST on two cancer types (IPMN and CRC) but does not explore how tissue-specific characteristics (e.g., stromal content, immune infiltration) might influence power estimation. Different tissues may have varying levels of spatial heterogeneity, which could affect the accuracy of power estimates.*

**Answer:** We thank the reviewer for this insightful comment. We agree that tissue-specific features and spatial heterogeneity can impact power estimation. In this study, we mainly focus on the carcinoma regions and the regions near it. This focus aligns with the design of the original IPMN study, which also did not analyze regions beyond the three included in our manuscript. We acknowledge that our current framework does not explicitly quantify the relationship between power and spatial heterogeneity. Instead, it relies on the assumption that the spatial correlation structure observed in the preliminary dataset is representative of the data to be collected in future experiments.

Such limitation has been clearly stated in the Discussion section (page 12, paragraph 2) of the revised manuscript:

PoweREST incorporates the spatial information of ST data by computing test statistics using the spot-level gene expression within the same ROI. Rather than imposing a fixed spatial correlation structure, our nonparametric approach adapts to complex spatial relationships within the ROI, offering a more accurate representation of the power surface. This modeling approach assumes that the spatial correlation observed in the preliminary dataset is reflective of that in future experiments. The ROI selection is typically guided by biological insights or tissue architecture with the help of biological investigators and pathologists. In this study, we focused on the carcinoma regions and their adjacent areas. However, alternative ROI selection criteria or study objectives may lead to different spatial characteristics, which in turn may influence the resulting power surface. In such cases, the estimated power may need to be adjusted to account for tissue heterogeneity, with the optimal sample size increased in the presence of greater heterogeneity or decreased when the target tissue is more homogeneous.

7. *As we know, cancer tissues demonstrate the high levels of spatial heterogeneity. The power estimation highly depends on tissue slice selections or region selections.*

**Answer:** We thank the reviewer for emphasizing this important point. Indeed, spatial heterogeneity is a hallmark of cancer, and we fully agree that tissue slice and ROI selection can impact power estimation results. The current design of PowerREST is based on real spatial transcriptomics data from CRC and PDAC patients, with regions of interest (ROIs) carefully selected by a pathologist to ensure high-quality input and minimize the influence of spatial heterogeneity and biological variability. However, we also acknowledge that no universally optimal strategy currently exists for addressing unpredictable spatial heterogeneity across tissues or conditions. Therefore, our framework assumes that the ROI selection strategy and spatial heterogeneity in the planned experiment are comparable to those observed in the pilot data used for modeling. We included

PoweREST incorporates the spatial information of ST data by computing test statistics using the spot-level gene expression within the same ROI. Rather than imposing a fixed spatial correlation structure, our nonparametric approach adapts to complex spatial relationships within the ROI, offering a more accurate representation of the power surface. This modeling approach assumes that the spatial correlation observed in the preliminary dataset is reflective of that in future experiments. The ROI selection is typically guided by biological insights or tissue architecture with the help of biological investigators and pathologists. In this study, we focused on the carcinoma regions and their adjacent areas. However, alternative ROI selection criteria or study objectives may lead to different spatial characteristics, which in turn may influence the resulting power surface. In such cases, the estimated power may need to be adjusted to account for tissue heterogeneity, with the optimal sample size increased in the presence of greater heterogeneity or decreased when the target tissue is more homogeneous.

in the Discussion section (page 12, paragraph 2)*.*

8. *PowerREST is primarily designed for power estimation in spatial transcriptomics, However, it is still unclear which parameter settings are most effective for capturing and leveraging spatial information. A more detailed discussion or analysis on how these parameters influence the power estimation, particularly in the context of spatial heterogeneity and tissue architecture, would be beneficial.*

**Answer:** We thank the reviewer for this valuable feedback. We agree that understanding how parameter choices affect power estimation is critical for practical use of PowerREST. In our framework, power is estimated based on three key variables: log-fold change, gene detection rate, and the number of slice replicates. Based on the XGBoost model, we evaluated the feature importance of the three input variables and visualized the results in Figure 5. The plot shows that mean_pct (average gene detection rate across slices) contributes the most to power prediction, followed by avg_log2FC_abs (average absolute log-fold change) and sample size. Our non-parametric simulation approach implicitly captures spatial structure by resampling spot-level gene expression data within defined ROIs. With a similar spatial structure between the simulated data and target data, the average gene detection rate and absolute log-fold change are the leading factoring impact the identification of differentially expressed gene signals.

**Figure 5**: Feature importance based on XGBoost’s result.

**Response to Reviewer #2’s comments:**

**Major**

*1.The authors stated PoweREST implicitly incorporates the spatial information as it samples spots within the same ROI. The spatial information might be utilized for detecting spatial clusters or defining ROIs, which is not part of the PoweREST framework. Power analysis tool designed for scRNA-seq data is likely to be able to achieve the same purpose, provided with properly defined ROIs/spatial clusters.*

**Answer:** We thank the reviewer for raising this important point. Firstly, the data structure of spatial transcriptomics differs substantially from that of scRNA-seq. Gene expression measurements in ST are typically sparser than scRNA-seq and the valuable spatial location information from ST does not exist in scRNA-seq. Secondly, to the best of our knowledge, most existing power calculation tools for scRNA-seq assume independence between the gene expression profiles of cells. In contrast, our method accounts for the spatial correlation of gene expression across difference cells within the same spot by employing the bootstrap sampling approach. Spatial correlation plays an important role in differential analysis between ROIs, as high correlation indicates more homogeneous gene expression within each ROI and can enhance statistical power. Existing scRNA-seq-based methods, which do not account for spatial structure, may therefore yield inaccurate power estimates when applied to ST data.

*2.Is PoweREST able to handle the situation where patient/donor-level difference exists (batch-effect, different number of replicates, imbalanced spot numbers in samples, etc) within each group? What would be the appropriate sampling strategy? Can PoweREST be used to calculate the minimum number of patients required to achieve desired statistical power in detecting DEGs?*

**Answer:** We thank the reviewer for raising these important and practical questions. We clarify that PoweREST can accommodate imbalanced study designs, including different numbers of replicates and imbalanced spot numbers across groups. However, we assume that the number of spots in the ROI is approximately consistent within each group. With this assumption, yes, PoweREST can be used to calculate the minimum number of patients required to achieve desired statistical power in detecting DEGs. We clarified these points by added

In this report, we assumed the number of spots and the number of replicates were equal across the two conditions. However, both assumptions can be relaxed. Specifically, PoweREST has the flexibility to accommodate imbalanced designs by allowing different numbers of replicates and different spot counts across experimental groups for power calculation. However, PoweREST still requires that the number of spots in the ROI is approximately consistent within each group.

in the Method section (page 4, lines 119-125).

Regarding batch effects, the IPMN and CRC data we currently worked with have been preprocessed and integrated to remove batch-effect, and thus our method does not consider batch information as a factor in our model system. This is based on the assumption that appropriate computational correction methods, such as Harmony^[1]^, can be applied during preprocessing to effectively mitigate batch effects. Our bootstrap sampling of spot-level gene expression also relies on the assumption that batch effects have been adequately removed during preprocessing. The datasets used in this study have already undergone such corrections by the data providers, ensuring that residual batch effects are minimal and unlikely to bias power estimation. We added

Our bootstrap sampling strategy relies on the assumption that batch effects have been adequately removed during preprocessing in earlier analysis. The datasets used in this study have already undergone such corrections by the data providers, ensuring that residual batch effects are minimal and unlikely to bias power estimation.

in the Discussion section (page 13, lines 410-414).

[1] Korsunsky, I., Millard, N., Fan, J., Slowikowski, K., Zhang, F., Wei, K., Baglaenko, Y., Brenner, M., Loh, P.R. and Raychaudhuri, S., 2019. Fast, sensitive and accurate integration of single-cell data with Harmony. Nature methods, 16(12), pp.1289-1296.

*3.Are there any other machine learning classification models able to impose multiple monotonic constraints on predictors? If so, it would be interesting to see their performance in comparison to XGBoost on local power estimation.*

**Answer:** We appreciate the reviewer’s interest in alternative machine learning models that support monotonicity constraints. While XGBoost is one of the most widely used and efficient models with built-in support for monotonic constraints on multiple predictors, there are a few other machine learning models. Upon reviewing current literatures, we found that machine learning models LightGBM^[1]^ and CatBoost^[2]^ also support monotonic constraints. And similar to XGBoost, both models are based on gradient boosting frameworks. We selected LightGBM for additional analysis due to its faster training speed, while CatBoost is specifically optimized for handling categorical features.

Though both XGBoost and LightGBM are based on gradient boosting frameworks, they differ in such a way that XGBoost expands all nodes at the same depth before moving deeper while LightGBM finds the leaf with the highest potential to reduce loss and only splits that leaf. We summarize their difference and hyperparameters in Table 9 (also available as S4 Table in Supplementary material). Despite differences in tree growth strategies, the results (Figures 6-7, also available as S8-S9 Figs in Supplementary material) are consistent with those obtained from XGBoost, indicating the robustness of our findings across gradient boosting implementations. We added

Apart from XGBoost, we also implemented LightGBM, another machine learning model based on gradient boosting framework, with monotone constraints applied. Although LightGBM and XGBoost differ in their tree construction strategies (summarized in S4 Table), the results from LightGBM are consistent with those obtained from XGBoost (S8-S9 Figs), indicating the robustness of our findings across gradient boosting implementations.

in the Results section (page 11, paragraph 2, lines 320-325).

**Table 9**: Comparisons between XGBoost and LightGBM decision tree growth strategies and hyperparameters.

|  | XGBoost | LightGBM |
| --- | --- | --- |
| How tree grows | Expand all nodes at one level before moving deeper | Expand only the best leaf |
| Tree shape | More balanced | More irregular and deep |
| Overfitting risk | Lower | Higher |
| **Hyperparameters** | | |
| Step size | eta | learning_rate |
| Max depth | max_depth | max_depth |
| Max number leaves | - | num_leaves |
| Number of parallel trees | num_parallel_tree | - |
| **Monotonic constrain** | **monotone_constraints** | **monotone_constraints** |

**Figure 6**: LightGBM results. (A) The fitted power surfaces for DE analysis within the carcinoma border with the number of slice replicates per group being 2, 4, and 6. (B) The relationship between the power and logFC with slice replicates ranging from 1 to 10 for the percentage of spots with the detected gene being 0.05, 0.10, and 0.15.

**Figure 7**: Comparison between results of LightGBM and XGBoost. (A) The absolute difference values between the fitting results obtained from LightGBM and XGBoost. (B) The feature importance of the fitted models. (C) The fitted XGBoost model. (D) The fitted LightGBM model.

C

D

[1] Ke, G., Meng, Q., Finley, T., Wang, T., Chen, W., Ma, W., Ye, Q. and Liu, T.Y., 2017. Lightgbm: A highly efficient gradient boosting decision tree. Advances in neural information processing systems, 30.

[2] Prokhorenkova, L., Gusev, G., Vorobev, A., Dorogush, A.V. and Gulin, A., 2018. CatBoost: unbiased boosting with categorical features. Advances in neural information processing systems, 31.

*4.Given the spot-level data, the DEG results might reflect shifts in cellular composition rather than transcriptional change, it would be helpful to incorporate this information into the framework.*

**Answer:** We thank the reviewer for this insightful observation. We fully agree that in spatial transcriptomics data, observed differential expression may arise from changes in cellular composition across conditions rather than from intrinsic transcriptional changes. This is a critical consideration when performing and interpreting DE analysis in spatial contexts^[1]^. Notably, the original publications associated with the datasets used in our study did not address this issue. We acknowledge this as a limitation and have now stated

Incorporating cellular compositions may further improve the power estimation, which can be our future research direction.

in the Discussion section (page 12, lines 373-375). And we are currently developing tools to address this problem.

[1] Li, Z. and Wu, H., 2019. TOAST: improving reference-free cell composition estimation by cross-cell type differential analysis. Genome biology, 20, pp.1-17.

*5.Can PoweREST be applied to high-resolution spatial transcriptomics data such as Xenium and CosMx?*

**Answer:** We thank the reviewer for raising this important point. While PowerREST was originally developed and validated using spot-based spatial transcriptomics data (10X Genomics Visium), the principles of our PoweREST methodology are applicable to other ST platforms such as Xenium and CosMx. To address this point, we have added

Though PoweREST is designed for 10X Genomics Visium platform and was evaluated on the visium data in this study, its non-parametric framework is inherently flexible and can be extend to other ST platforms, as its simulation-based approach does not assume platform-specific parametric distributions. In future work, we plan to generalize PoweREST to support other ST platforms.

in the Discussion section (page 12, lines 369-373).

**Minor**

1. *In line 118, “ie” should be “i.e.”.*

**Answer:** Thank you for pointing this out. We have corrected “ie” to “i.e.” in (page 4, line 119, originally line 118) of the revised manuscript.

2. *In Figure 5A, the axis labels are mismatched.*

**Answer:** We thank the reviewer for catching this oversight. We have corrected the axis labels in Figure 5A. The updated figure has been included in the revised manuscript.

**Figure 8**: Revised version of Figure 5A.

3. *In line 289, the authors mentioned that they experimented with XGBoost using a larger range of logFC and detection rate. However, the result in Figure S3 comes from the same range. The numbers of the pct axis in Figure S3A are hard to read.*

**Answer:** We appreciate the reviewer’s careful reading and helpful feedback. We would like to clarify that we did indeed run XGBoost based on the full range of logFC and detection rate values, as stated. However, in the original version of Figure S3A (now S7 Fig), the plot was cropped for visualization purposes. To avoid confusion, we have now revised it (Figure 9) to display the full range of input values and have also improved the readability of the pct axis labels by providing a top view.

**Figure 9**: Estimation of the full power surface using XGBoost (A) .

4. *The authors mentioned that the bootstrap-based method could be computationally expensive. What's the runtime for sampling and model fitting implemented on the data shown in the manuscript?*

Answer: Thank you for the question. The power calculation in the manuscript was executed on a high-performance computing cluster using 12 CPU cores and a memory allocation of 56 GB. We used presto library^[1]^ via Seurat's *FindMarkers()* to run fast DE analysis and found that in this way the run time is mainly determined by the times of bootstrap sampling rather than the replicates per group (Figure 10, also available as S10 Fig in Supplementary material). Users can also use our shiny app which takes less than 2s to generate the results.

In the Discussion section (page 13, lines 399-407), we added

The power calculation in the manuscript was executed on a high-performance computing cluster using 12 CPU cores and a memory allocation of 56 GB. One potential disadvantage of our bootstrap-based approach is the heavier computational burden. We recommend users carry out the simulation using the presto library to run fast DE analysis. The bar plot (S10 Fig) shows the runtime versus the number of bootstraps used in an analysis, stratified by number of replicates per group. It was found that the run time is mainly determined by the times of bootstrap sampling rather than the replicates per group and at 100 bootstrap times, the runtime is about 78s. We also provided the pilot results of power estimations for DE analysis in the PoweREST R Shiny app for two different tissue types which takes less than 2s to generate the results. In the future, we aim to upload power estimation results for more cancer types.

**Figure 10**: Runtime of PoweREST

[1] Korsunsky, I., Nathan, A., Millard, N., & Raychaudhuri, S. (2019). Presto scales Wilcoxon and auROC analyses to millions of observations. bioRxiv, 653253. https://doi.org/10.1101/653253
